# Cultural adaptation of mental health first aid guidelines for depression for Sri Lanka: a Delphi expert consensus study

**DOI:** 10.1186/s12888-021-03598-0

**Published:** 2021-11-20

**Authors:** Madhawee Fernando, Amila Chandrasiri, Madhubhashinee Dayabandara, Nicola J. Reavley

**Affiliations:** 1 Centre for Mental Health, Melbourne School of Population and Global Health, University of Melbourne, Parkville, Victoria 3010 Australia; 2 Ministry of Health, Colombo, Sri Lanka; 3 Department of Psychiatry, Faculty of Medicine, University of Colombo, Colombo, Sri Lanka

**Keywords:** Depression, Mental health first aid (MHFA), Cultural adaptation, Delphi study, Sri Lanka

## Abstract

**Background:**

Family and friends can play a key role in supporting a person with depression to seek professional help. However, they may lack the knowledge to do so. English-language guidelines for high-income countries have been developed to assist with this. The aim of this study was to adapt the English mental health first aid guidelines for helping a person with depression to the Sri Lankan context.

**Methods:**

A Delphi expert consensus study involving mental health professionals and people with lived experience (either their own or as carers) was conducted. Participants were recruited from inpatient, outpatient and community care settings. The English-language questionnaire was translated into Sinhala and participants were asked to rate the importance of each item for inclusion in the guidelines for Sri Lanka.

**Results:**

Data were collected over two survey rounds. A total of 115 panellists (23% male) consisting of 92 mental health professionals and 23 consumers and carers completed the Round 1 questionnaire. A total of 165 items were included in the final guidelines, with 156 adopted from the guidelines for English-speaking countries and 9 generated from the comments of panellists.

**Conclusions:**

The adapted guidelines were similar to the English-language guidelines. However, new items reflecting culturally relevant approaches to autonomy-granting, communication and culture-specific manifestations of depression were reflected in the adapted version. Further research should explore the use of the adapted guidelines, including their incorporation into Mental Health First Aid Training.

**Supplementary Information:**

The online version contains supplementary material available at 10.1186/s12888-021-03598-0.

## Background

Depression is the most prevalent mental disorder globally, affecting more than 264 million people [1]. The incident cases of depression worldwide increased by 49.86% from 1990 to 2017, pointing to the expanding scale of this public health problem [2]. Depression carries burdensome societal costs and has been linked to impaired functioning in societal roles (e.g. low marital quality, low work performance, low earnings), severity of a wide range of chronic physical illnesses and increased mortality due to physical illness and suicide [3]. Given these wide-ranging societal costs, depression has been identified as a barrier to sustainable development across all regions of the world [4]. A treatment gap exists in low- and middle-income countries whereby a large proportion of individuals experiencing depression do not have access to evidence-based treatments due to a lack of available mental health services and limited mental health literacy of the population (Patel et al., 2018).

A number of population-based studies to assess prevalence of depression in Sri Lanka have been conducted, A national mental health survey of over 6000 participants conducted in 2007 found a prevalence estimate, as measured by the Patient Health Questionnaire-9 (PHQ-9), of 6.9% for mild to moderate depression and 2.4% for major depression [5]. A population-based study conducted in Colombo in 2010 reported a 6.6% lifetime prevalence estimate of depression rising to 11.2% if the functional impairment criterion was excluded [6]. A study conducted with a Sri Lankan undergraduate student sample showed that approximately 10% reported a diagnosis of Major Depressive Disorder (MDD) and 20% screened positive for depression using the Sinhala and Tamil versions of the (PHQ-9) [7]. However, much higher prevalence rates have been seen in respondents recruited from hospital inpatient and outpatient settings [8–10] and people with comorbid chronic illness [11, 12], samples of adolescents [13] and older adults [14].

A recent systematic review that captured 77 nationalities and ethnicities suggests that the experience of depression and symptom expression may not be uniform across different regions of the world [15, 16]. For example, in the South-Asian region depression is commonly expressed as a range of somatic symptoms and complaints [17, 18] suggesting the need for culturally adapted approaches to assessment [18] and treatment of depression [19].

In Sri Lanka, stigmatising attitudes and poor mental health literacy about depression are ongoing obstacles to help seeking. A survey of carers of those living with depression showed that 61% held the attitude that depression was a sign of personal weakness and 60% believed that a person living with depression is more violent than a member of the general community [20]. Carers in this study also endorsed dealing with depression alone [20]. A study conducted with undergraduate students, in which respondents could answer in English or Sinhala, suggested that only 17.4% recognized depression, with a significantly lower recognition rate among those responding in Sinhala (3.5%) [21]. Depression recognition in this Sri Lankan study was considerably lower compared to survey studies in western countries where up to 75% participants aged 15 and over were able to recognise depression [22]. However, the ability to recognise the problem as depression was associated with the likelihood of endorsing helpful treatment. Similarly, undergraduates’ ability to recognise depression was the strongest predictor of their intention to seek help while stigmatising attitudes towards people with depression decreased this likelihood [23]. A study that explored helping intentions of undergraduate students towards a peer with depression showed that only one third considered the need for professional help [24] and that help seeking intentions were lower among those with personal experiences of depression problems and those who perceived depression as weakness. These findings point to the need for interventions to enhance mental health literacy about depression to encourage help-seeking and support for those living with depression in Sri Lanka.

Mental Health First Aid (MHFA), a training course for members of the public on how to assist someone developing a mental illness or mental health crisis situation (e.g., suicidal intent or traumatic experience), until the person receives professional help or the crisis resolves [25] has the potential to assist in meeting this need. MHFA was first developed in Australia in 2000 and by mid-2018, had trained over 700,000 [26]. Australians. MHFA training has now spread to over 25 countries reaching over four million people globally and  has shown demonstrable success in improving mental health first aid knowledge, recognition of mental disorders, beliefs about effective treatment and reduction in stigma up to 6 months after initial training [27, 28].

Studies have suggested cross-cultural generalizability of Mental Health First Aid guidelines but that some culturally specific adaptations may be needed [29]. A recent adaptation of the mental health first aid guidelines for depression to the Chinese context suggests that, even though the overall guidelines remained similar to those for English-speaking countries, new actions relating to ways of respecting the autonomy of a person living with depression and the role of families were included [30]. MHFA guidelines for helping a person at risk of suicide have been adapted to the Sri Lankan context in English language only [31] and in Sinhala [32] and suggests that even though adapted guidelines are similar to the English guidelines, cultural adaptations were needed around family involvement and explicit mention of suicide. This suggests the need for specific tailoring of the MHFA guidelines on depression to the Sri Lankan context as well.

Therefore, the aim of the study was to use the Delphi expert consensus method with English and Sinhala-speaking Sri Lankan mental health professionals and consumers to culturally adapt the Mental Health First Aid guidelines for depression for Sri Lanka.

## Methods

The Delphi methodology, which is a systematic method for establishing expert consensus on a topic, was used to achieve consensus on potential statements to be included in the mental health first aid guidelines for depression in Sri Lanka. This method is widely used in mental health research and allows researchers to access the cumulative experience and judgement of a larger body of experts on a particular topic [33].

The current Delphi consensus study involved four stages 1) Questionnaire development for Round 1 by translating questionnaires used to develop the English language guidelines into Sinhala. 2) Panel identification and recruitment 3) data collection and analysis over 2 rounds of survey 4) guideline development.

### Questionnaire development for round 1 of Delphi survey

The mental health guidelines for depression [34] were translated into Sinhala by a health professional. Some of the translated statements were then modified to ensure that they were more appropriate to the Sri Lankan health system and cultural context. The round 1 questionnaire comprised 175 items under 8 headings (see Table 1). The questionnaire also contained questions about socio-demographic characteristics, professional status and experience in mental health service provision (for health professionals). At this stage, questionnaires were only translated into Sinhala as this is the most widely spoken official language of Sri Lanka. The final stage will involve translating the guidelines into Tamil.

**Table 1:** Sections and the number of statements included in each round

Guidelines section	Statements rated in Round 1 (n)	Statements rated in Round 2 (n)	Total included (n)
*Section 1: How do I know if someone is experiencing depression* -Learning about depression -If the first aider notices signs or symptoms of depression -Preparing for the conversation -Having a conversation -Giving the person information	35	4	32
*Section 2: How can I be supportive* -Treat the person with respect and dignity -Do not blame the person -Expectations -Offering consistent support and understanding -What doesn’t help -Give the person hope for recovery	47	6	41
*Section 3: Communicating effectively* - Encourage the person to talk -Be a good listener	31	3	29
*Section 4: Difficulties the first aider may encounter*	21	2	19
*Section 5- Cultural considerations*	3	0	3
*Section 6: Encouraging help-seeking* -When to encourage help-seeking -How to assist the person with help-seeking -Self-help strategies	24	0	22
*Section 7: What to do if the person doesn’t want help*	7	1	5
*Section 8: Concerns for safety*	6	1	5
New items	NA	12	9
**Total**	**175**	**29**	**163**

### Panel identification and recruitment

#### Eligibility

Participants were recruited into one of two panels, one comprising mental health professionals and the other comprising consumers (people with lived experience of depression) and carers. Mental health professionals were eligible to participate if they had been providing mental health services for at least 2 years in either public or private sectors in a curative or preventative capacity.

Consumers or carers were eligible to participate if they met the following criteria:

1. They had at least 1 year’s lived experience after the diagnosis of depression or
2. They had at least 1 years’ experience providing care to a person living with depression.

#### Sampling methods

Participants were selected using purposive and snowball sampling methods. With the goal of capturing a diverse range of opinions, multiple recruitment sites were identified across administrative districts of Sri Lanka and spanning primary, secondary and tertiary level care, inpatient, outpatient and community care. Approval was obtained from relevant administrative authorities to approach participants at these sites. One of the authors (AC), a community medicine practitioner, visited the different sites during monthly review meetings, explained the purpose of the study and recruited mental health professionals directly.

Consumers were recruited with the help of a study coordinator (typically a clinical nurse) at each site. Coordinators explained the purpose of the study and invited consumers who were eligible and interested to a session in which the questionnaires were administered.

The research was approved by the Human Research Ethics Committee at the University of Melbourne (HREC No.1750853.1) and Ethics Review Committee of Faculty of Medicine, University of Colombo.

### Data collection and analysis

Participants were instructed to rate how important each statement was to be included in a set of guidelines for providing mental health first aid to a person experiencing depression. Each statement was rated on a five-point scale with the following options: Essential, Important, Don’t know/Depends, Unimportant, Least Important. In round 1, panelists were also encouraged to make comments modifying existing statements or to suggest other helpful actions that had not been covered in the questionnaire. Mental Health Professionals were offered the choice of completing the questionnaires in either English or Sinhala while consumers were offered the Sinhala version of the questionnaires. Participants were reimbursed for their time with a gift voucher of 1500 Sri Lankan rupees.

Statements were immediately included in the guidelines if they were endorsed as “Essential” or “Important” by ≥80% of both panels. Statements were re-rated in the subsequent round if they were rated as either essential or important by 70–79% of either panel. Statements were immediately excluded from the guidelines if they were rated as essential or important by less than 70% of either panel.

Comments and suggestions from participants were collected, sorted and translated into English by one of the authors (AC) and then reviewed by the authors NR, AC and MF. New ideas were written into statements and included in the Round 2 questionnaire. The Round 2 questionnaire comprised 17 items selected for re-rating based on the aforementioned criteria and 12 new items. Statements were re-rated using the five-point Likert scale as above. Statements were included after Round 2 if they were rated above 70% by both panels (a threshold lower than the 80% used in studies that use three survey rounds, primarily due to the need to use paper rather than online questionnaires).

The correlation between endorsement rates of the two panels of professionals and consumers were measured by the Spearman’s correlation coefficient using SPSS version 25.

### Development of the guidelines

Endorsed statements (i.e those rated as either essential or important by ≥80% of both panels) in both rounds were compiled. Author MF drafted the guidelines by writing the list of endorsed statements into sections of connected text. Where possible statements were combined in order to minimize repetition and enhance flow of text. The draft was circulated to a panel of Sinhala-speaking mental health professionals for final review.

## Results

### Expert panel formation

A total of 115 panellists (23% male) consisting of 92 mental health professionals and 23 consumers and carers completed Round 1 of this Delphi study. The demographic characteristics of the participants are shown by panel in Table 2.

**Table 2:** The socio-demographic characteristics of all participants

Variable	Mental health professionals		Consumers	
	Frequency (*n* = 92)	Percentage (%)	Frequency (*n* = 23)	Percentage (%)
Gender				
Male	21	22.8%	6	26.1%
Female	71	77.2%	13	56.5%
Missing	0	0%	4	17.4%
Age category				
18–34	22	23.9%	4	17.4%
35–44	33	35.9%	4	17.4%
45–54	33	35.9%	2	8.7%
55–64	4	4.3%	5	21.7%
65 and above	0	0%	4	17.4%
Missing	0	0%	4	17.4%
Highest educational qualification				
Primary school	0	0%	4	17.4%
Secondary school / high school	5	5.4%	13	56.5%
Technical diploma	48	52.2%	1	4.3%
Bachelor’s degree	19	20.7%	0	0%
Master’s degree	4	4.4%	0	0%
Doctorate (Higher degree by research) or PhD	6	6.5%	0	0%
Other	10	10.9%	0	0%
Missing	0	0%	5	21.7%
Principal area of practice				
Psychiatrist	8	8.7%	NA	NA
Other medical doctors providing mental health services	18	19.6%	NA	NA
Midwife providing preventative mental health services	22	23.9%	NA	NA
Mental health nurse	33	35.9%	NA	NA
Mental health social worker	2	2.2%	NA	NA
Other	8	8.7%	NA	NA
Missing	1	1.1%	NA	NA
Principal setting of practice / affiliation				
Government hospital	58	63%	NA	NA
Community mental health service	28	30.4%	NA	NA
Educational institution	1	1.1%	NA	NA
Other	3	3.3%	NA	NA
Missing	2	2.2%	NA	NA
Years worked in the principal area of practice/ as consumer				
2–4 years	31	33.7%	4	17.4%
4 years or more	58	63.%	19	82.6%
Missing	3	3.3%	0	0%
Consumer/carer status				
Consumers with lived experience	NA	NA	18	78.2%
Carers	NA	NA	5	21.7%

A higher number of females participated in the study across both the health professional (77.2%) and consumer (56.5%) panels. The majority of mental health professionals were aged 35 to 54 (71.7%), nurses or midwives (35.9 and 23.9% respectively) and affiliated with government hospitals (63%) or community mental health services (30.4%). In addition, 26 doctors involved in the provision of mental health services, including 8 psychiatrists, participated in the study. Allied health services were under-represented in the sample which comprised 2 mental health social workers (2.2%) but no psychologists or occupational therapists. Most health professionals had over 4 years of experience providing mental healthcare (63%).

Of the 23 consumers who participated in the study, 18 had personal experience of depression while 5 had experience providing care for someone living with depression. The majority of consumers were female (56.5%) had a secondary school level education (56.5%) and had been living with depression or caring for someone with depression for over 4 years (82.6%). Among the mental health professionals who took part in the first round, 72 (78%) were retained in round 2, while the retention rate among consumers was 39.1% (*n* = 9).

### Ratings

**Fig. 1:**
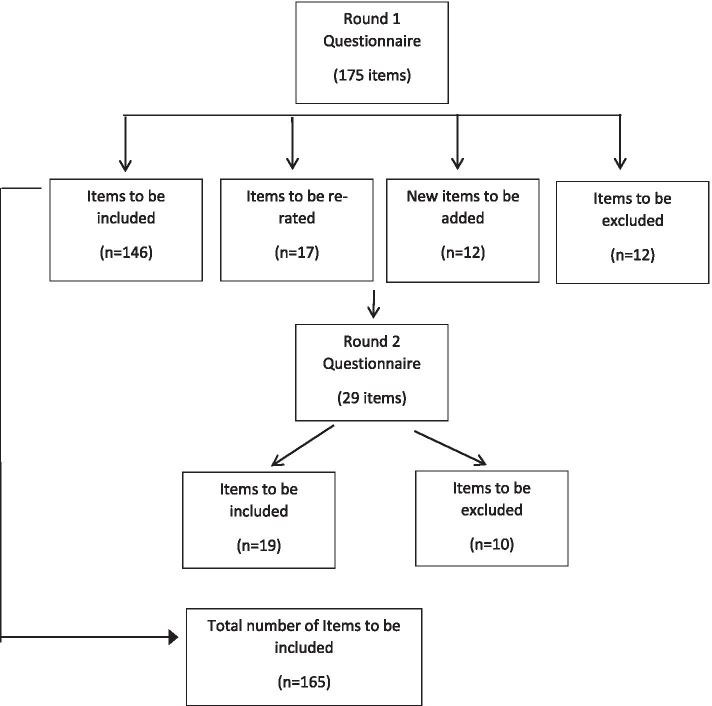
Overview of study rounds

Of the 175 statements translated from the English guidelines, 146 were endorsed by over 80% of both panels in round 1, 17 were re-rated and 12 were omitted. A further 12 statements were developed from panelists’ comments and a total of 29 items were rated in round 2. At the end of round 2, items with an endorsement rate of less than 70% from either panel (*n* = 10) were omitted (7 items from the guidelines translated from English and 3 newly developed items) and the rest (*n* = 19) were included in the final guidelines. The final version of the guidelines included 165 endorsed statements (See Fig. 1). A complete list of items and ratings is provided in Additional file 1. The Spearman’s correlation coefficient between the final statement endorsement rates of the two panels was 0.38 in Round 1 (*P* < 0.001).

### Differences between the English and Sinhala guidelines

The guidelines endorsed by the Sri Lankan expert panels were largely similar to the guidelines for English-speaking countries (156 items from the original guidelines were included in the Sri Lankan version). Omitted items covered aspects of language use and respecting a person’s autonomy and right to reject professional help. The nine items that were newly developed for the Sri Lankan context provide more specific guidance around helpful ways to approach a conversation with a person living with depression in a gentle and culturally appropriate way (e.g., culture-specific manifestations of depression, involvement of the family and self-help).

## Discussion

This research aimed to culturally adapt the Mental Health First Aid Guidelines for depression currently used in English-speaking countries to the Sri Lankan context. To this end, a Delphi consensus study was conducted with 92 Sri Lankan mental health professionals and 23 consumers and carers. The final set of guidelines consisted of 165 endorsed statements in total.

### Comparison with the guidelines for English-speaking countries

Overall, a large proportion of the guidelines originally used in English speaking countries (89%, 156 out of 175 statements) was included in the culturally adapted Sri Lankan version, indicating broad agreement between Sri Lanka and English-speaking countries on ways to provide mental health first aid to someone living with depression. However, there were some notable differences. Ten of the statements in the original guidelines were excluded and nine new statements introduced. Several key issues related to cultural adaptation to Sri Lanka were noted.

Statements related to respect for a person’s autonomy such as respecting the person’s right to reject help, respecting the person’s feelings, personal values and experiences as valid even if the first aider disagrees with them and not pushing the person to do activities that they may feel are too much for them were not endorsed. In a collectivist cultural context such as Sri Lanka, a first aider may be expected to prioritise a person’s health, safety and functioning over respect for their autonomy [35]. In the same vein, family involvement was emphasized in a new statement that was added (e.g., If the first aider is not a family member, they should ask family members of the person about their symptoms). In Sri Lanka, individuals are strongly embedded in their family networks and rely on family for care and support in recovery [36]. Therefore, family involvement appears to be assumed rather than seen as a choice [37]. These additions are similar to those seen in the cultural adaptation of the English-language guidelines for China [30].

The importance of giving the person hope for recovery and letting them know that their life is important was highlighted in the original guidelines. In the Sri Lankan version, a new item was endorsed around reminding the person of their valuable role in society (e.g., The first aider should tell the person that they have a valuable role in society). This may further reflect a collectivist world-view whereby a person’s sense of worth or value is related to their role and contribution to society [38].

A new item around the importance of understanding culturally specific manifestations of depression was included after a panellist’s suggestion. This is consistent with previous research that suggests that in South Asia, depression may present as a range of somatic complaints [15, 18].

Certain statements from the original guidelines related to discourse style, language use and non-verbal cues were omitted. For example, guidelines on maintaining an open body posture and not using patronising language or overly compassionate looks of concern were not endorsed. Newly added items provided culturally-appropriate ways of approaching the topic of depression (e.g., If the first aider thinks someone may be depressed, they should try to start the conversation by talking about neutral topics, e.g., day-to-day life issues or topics in common) and guidance on not forcing the conversation (e.g., “the first aider should let the person know that if they don’t want to talk, they are happy to do this at another time). Previous research suggests that when the communication style is not perceived as culturally sensitive, the impact of health messages and patient satisfaction are reduced [39]. Therefore, the inclusion of elements related to communication style may enhance the likelihood that mental health first aid is accepted.

### Strengths and limitations

A notable strength of the current study was that it combined a well-established, evidence-based approach to the provision of mental health first aid with a systematic approach to cultural adaptation. This allowed for cultural differences in attitudes towards autonomy, communication styles and culture-specific manifestations of symptoms to be reflected in the adapted guidelines. Moreover, a large number of mental health professionals (*n* = 92) spread across multiple geographical districts participated in the study.

Even though the study adhered to the minimum recommended number of experts for a Delphi study [40], due to recruitment difficulties, a smaller number of consumers and carers participated in the study (n=23) compared to mental health professionals (n=92) and the correlation between final statement endorsement rates was also lower than that seen in other cultural adaptation studies [30], possibly due to lower mental health literacy in the general population in Sri Lanka [5, 21]. Other limitations include the lack of involvement of consumer advocacy organisations and also that allied health professionals may be under-represented in the sample. Allied health professionals’ involvement in mental health service provision is an emerging area within the Sri Lankan context and the current study may not have captured their perspective adequately. Comparisons with the English-language guidelines are limited by potential differences in understanding terminology in English and Sinhala. However, as the guidelines are not clinical guidelines and do not use highly technical language, and the non-health professional participants were able to answer the questions or to make comments where items were unclear, we don’t believe this to be a significant limitation. Finally, the guidelines were not translated into Tamil (the second official language of Sri Lanka) during the Delphi study stage and the endorsed items may not represent the perspectives of Tamil speakers.

### Considerations for future use of the adapted guidelines

The culturally adapted guidelines will be made available as a stand-alone document and also used to inform the development of an MHFA training manual and curriculum. Translation of the guidelines into Tamil and inclusion of evidence-based culturally relevant information such as symptoms of depression [18] are important considerations in the implementation process given that overcoming linguistic discrimination and language-based disparities in access to resources is an ongoing priority in post-conflict Sri Lanka [41].

There is also a need to further explore ways in which the adapted guidelines and training may be disseminated in the Sri Lankan context across healthcare, education and community settings. Given previous research, carers of those living with depression and university students may be an important target of initial intervention as gaps in knowledge and sigma were identified.

In the Australian context, MHFA has reached over 2% of the population and there is evidence that training is associated with improved helping behaviours [26]. If similar dissemination outcomes were seen in Sri Lanka, population level increases in depression literacy and reduction in stigma could be achieved.

## Conclusions

The adapted guidelines were largely similar to the guidelines used in English-speaking countries, however, culturally relevant approaches to autonomy-granting, communication and culture-specific manifestations of depression were reflected in the adapted version. The adapted guidelines can be used as a stand-alone intervention by those seeking to provide mental health first aid to individuals in their social networks. At a community level, the guidelines have the potential to enhance public knowledge around mental health first aid for depression, skills for early recognition of depression and beliefs about effective treatment, as well as combat stigma around depression.

## Supplementary Information

**Additional file 1.**

## Data Availability

The datasets used and/or analysed during the current study available from the corresponding author on reasonable request.
